# RNA Viruses, Pregnancy and Vaccination: Emerging Lessons from COVID-19 and Ebola Virus Disease

**DOI:** 10.3390/pathogens11070800

**Published:** 2022-07-15

**Authors:** Chandrasekharan Rajalekshmi Dhanya, Aswathy Shailaja, Aarcha Shanmugha Mary, Sumodan Padikkala Kandiyil, Ambili Savithri, Vishnu Sasidharan Lathakumari, Jayakrishnan Therthala Veettil, Jiji Joseph Vandanamthadathil, Maya Madhavan

**Affiliations:** 1 Department of Biochemistry, Government College Kariavattom, Thiruvananthapuram 695581, India; dhanyasbabu@gmail.com; 2 Department of Pediatrics, Duke University School of Medicine, Durham, NC 27710, USA; aswathy.shailaja@duke.edu; 3 Department of Microbiology, School of Life Sciences, Central University of Tamil Nadu, Thiruvarur 610105, India; aarcha17@students.cutn.ac.in; 4 Post Graduate Department of Zoology, Government College, Vadakara 673102, India; sumodanpk@gmail.com; 5 Department of Biochemistry, Sree Narayana College, Kollam 691001, India; ambilisnc@gmail.com; 6 Department of Biochemistry and Industrial Microbiology, Sree Narayana College for Women, Kollam 691001, India; vishnusl@sncwkollam.org; 7 Department of Zoology, Government Brennen College, Thalassery 670106, India; jkbrennen@brennencollege.ac.in (J.T.V.); jijijoseph@brennencollege.ac.in (J.J.V.); 8 Department of Biochemistry, Government College for Women, Thiruvananthapuram 695014, India

**Keywords:** COVID-19, Ebola, immune response, vaccine, pregnancy, SARS-CoV-2, RNA virus, lymphopenia, T-cell exhaustion

## Abstract

Pathogenic viruses with an RNA genome represent a challenge for global human health since they have the tremendous potential to develop into devastating pandemics/epidemics. The management of the recent COVID-19 pandemic was possible to a certain extent only because of the strong foundations laid by the research on previous viral outbreaks, especially Ebola Virus Disease (EVD). A clear understanding of the mechanisms of the host immune response generated upon viral infections is a prime requisite for the development of new therapeutic strategies. Hence, we present here a comparative study of alterations in immune response upon SARS-CoV-2 and Ebola virus infections that illustrate many common features. Vaccination and pregnancy are two important aspects that need to be studied from an immunological perspective. So, we summarize the outcomes and immune responses in vaccinated and pregnant individuals in the context of COVID-19 and EVD. Considering the significance of immunomodulatory approaches in combating both these diseases, we have also presented the state of the art of such therapeutics and prophylactics. Currently, several vaccines against these viruses have been approved or are under clinical trials in various parts of the world. Therefore, we also recapitulate the latest developments in these which would inspire researchers to look for possibilities of developing vaccines against many other RNA viruses. We hope that the similar aspects in COVID-19 and EVD open up new avenues for the development of pan-viral therapies.

## 1. Introduction

RNA viruses pose the greatest threat to public health, with the potential to cause global catastrophic biological events, necessitating the identification of attributes of these microorganisms so as to open up new therapeutic and prophylactic avenues. In recent times, we have come across many viral outbreaks, which put vulnerable individuals at high risk but differ in the vectors of transmission, rates of fatality and transmissibility. Certain viruses such as Dengue and Zika require an intermediate host for their transmission, while diseases such as COVID-19 and Ebola Virus Disease spread directly from human to human. COVID-19, which resulted in the highest number of deaths globally in the recent past, has triggered a lot of research into the mechanisms of immune responses generated by RNA viruses and also into the various approaches for combating such viral outbreaks. The experiences with the Ebola outbreak in West Africa have provided valuable lessons to the global community in shaping the initial and quick management strategies for COVID-19.

A thorough investigation of the latest findings on the host antiviral immune responses in COVID-19 and EVD (Ebola Virus Disease) would be supportive in planning future research on pandemic/epidemic pathogens, and hence we summarize the current status in this direction. The high morbidity and mortality presented by most diseases due to RNA viruses call for intensive research into the development of vaccine technologies against them. Hence, we present here the latest update on the vaccines being developed against COVID-19 and EVD and the immune response generated by them. Another aspect that, we think, needs to be addressed is the status of pregnant women in relation to infection with RNA viruses. Pregnancy, alone, being associated with a state of immune alterations, exposes the maternal immune system to many challenges. Pregnant women, being a highly vulnerable group, need to be administered vaccines as early as possible; however, there is a lot of vaccine hesitancy among the population regarding immunization of pregnant women, who are avoided in the initial phases of most clinical trials. Hence, a look at available studies on the mechanisms of immune response in pregnant women and further, the reports of vaccine efficacy and outcomes among pregnant women against COVID-19 and EVD would definitely yield many insights that could be useful in the surveillance and planning of vaccination strategies for pregnant women against impending pathogenic RNA viruses.

## 2. A Comparative Look at the Pathogenic RNA Viruses—SARS-CoV-2 and Ebola Virus

The causative pathogen for the COVID-19 global pandemic is SARS-CoV-2, belonging to the family of Coronaviruses which are positive-stranded RNA viruses. Since elucidation of the molecular profile of the virus, the evolution of several variants of concern has occurred [1]. In contrast, there is comparatively more literature available for Ebola virus disease (EVD) also known as viral hemorrhagic fever. EVD caused by Ebola virus (EBOV) that belongs to the Filoviridae family which comprises six species, of which Zaire Ebola virus, Bundibugyo virus and Sudan virus have caused huge human outbreaks [2], among which the Zaire Ebola virus is found to be the deadliest.

Most of the emerging RNA viruses capable of infecting humans are found to be of zoonotic nature with known or suspected reservoirs in animals. COVID-19 is believed to originate from the outbreak of SARS-CoV-2 which started in Wuhan, China, probably by a spillover event from an unknown animal host [3]. In the case of EBOV, wild animals, especially fruit bats are the natural reservoirs [4], likely spreading to humans upon contact and subsequent entry following breaks in the mucosal surfaces. Further spread occurs as a result of direct contact with the patients and their body fluids or through nosocomial transmission. While in the case of COVID-19, the mode of transmission is primarily through respiratory droplets and aerosols [5]. Though the rate of transmission is much higher for COVID-19, the mortality rates are greater for EVD.

Both these RNA viruses have an incubation period before the onset of symptoms. Filoviral infection is followed by non-specific flu-like symptoms similar to the prodrome of many other viral diseases. In a retrospective study conducted in non-severe cases of COVID-19, four phases of the disease course have been proposed: a prodromal phase in the first week followed by a manifestation phase in the second week and finally a convalescent phase after three weeks of infection [6]. Disease progression of EBOV is characterized by maculopapular rashes and the late stage of the disease is indicated by complications of the gastrointestinal, respiratory, vascular and neurological systems, multi-organ failure and subsequent death [7]. Multi-organ damage followed by death is also a characteristic feature encountered in severe COVID-19 [8].

One of the strategies used by RNA viruses is to condense their genetic information into small genomes, which are translated immediately after viral entry into proteins needed for replication. EBOV has a single linear negative sense RNA of 19kb size, that codes for nucleoprotein (NP), polymerase cofactor VP35, matrix proteins VP40 and VP24, glycoprotein (GP), transcription activator VP30, and an RNA-dependent RNA polymerase (L) [7,9,10,11,12]. However, SARS-CoV-2 virus carries one of the largest genomes among RNA viruses, with a size of approximately 30 kb (almost double the size of EBOV genome) [13]. Two large polyproteins pp1a and pp1ab are formed by translation of the ORF, which in turn lead to production of functional viral RNA polymerase. The four structural proteins of SARS-CoV-2 virus have been well-characterized—Spike (S), Membrane (M), Envelope (E) and Nucleocapsid (N). The spike protein has a receptor binding domain (RBD) which is poorly conserved and is a highly specific target for human antibodies [14]. Both SARS-CoV-2 and EBOV use a surface glycoprotein for viral entry into the host cell, which then binds to a specific receptor on the host cell, leading to endocytic fusion of the virus. SARS-CoV-2 utilizes the ‘S’ protein [15] and EBOV makes use of Glycoprotein (GP) [16,17,18,19] for viral entry. The primary host receptor used, in the case of SARS-CoV-2, is ACE-2 which is highly expressed in alveolar and intestinal epithelial cells [20], while in the case of EVD, the primary host receptor is T-cell immunoglobulin and mucin domain-1 (TIM-1) [21,22]. In both cases, the viral entry is followed by replication of the viral genome and propagation of new viral particles that takes place through a series of successive steps. Structural aspects and mechanisms of pathogenesis associated with SARS-CoV-2 and EBOV have been compared in Table 1.

The role of lipids in viral entry and pathogenesis has been a hot topic of research. The involvement of lipids is proposed to occur mainly at three levels—the interaction of viruses with the lipid barrier offered by the host cell membrane, regulation of lipid metabolism to fuel viral replication and stimulation of production of several lipid mediators which can modulate the host immune response. Most enveloped viruses that have a mechanism of endosomal escape make use of apoptotic mimicry when Phosphatidylserine/Phosphatidylserine receptors serve as a route of virus entry. Phosphatidylserine (PS) receptors are demonstrated to facilitate infection of SARS-CoV-2 and hence have been suggested as a therapeutic target [37]. EBOV is also known to make use of the host apoptotic clearance mechanism to augment its entry into target cells by externalizing PS on its surface [38,39,40]. In both SARS-CoV-2 [37] and EBOV [41,42], the T cell Ig and mucin domain (TIM) family of proteins is shown to be involved in the above mentioned mechanism. Lipid raft domains rich in cholesterol and sphingolipids, on cellular membranes that play a significant role in viral trafficking and pathogenicity, are also studied in SARS-CoV-2 [43] and EBOV Virus [44].

## 3. Host Anti-Viral Immune Response in SARS-CoV-2 and EBOV Infections

Antiviral immunity in humans is comprised of a rapid nonspecific innate response intended to eliminate the virus, followed by B- and T-cell-mediated virus-specific adaptive responses [45]. In the ensuing sections, we have presented a comparison of the host immune response mechanisms involved in COVID-19 and EVD which would facilitate similar studies on diseases caused by other RNA viruses.

### 3.1. Defining Innate Immunity

**Figure 1:**
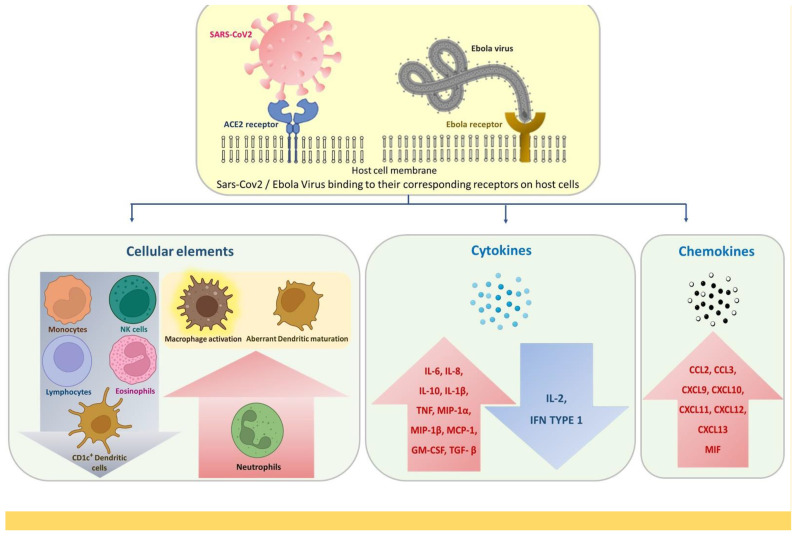
Innate immune response alterations common for SARS-CoV-2 and EBOV infections.

Innate immune responses hold the first line of defense against the attack of any microbial agent. Pathogen associated molecular patterns (PAMPS) derived from the pathogen, recognized by pattern recognition receptors (PRR) alert the innate immune system to the infection. This is followed by release of cytokines which then trigger production of pro-inflammatory molecules resulting in formation of a pro-inflammatory feedback loop [46]. This mechanism has been proposed for SARS-CoV-2 [46] and EBOV [47], which in turn leads to a hyperinflammatory immune response known as Cytokine Storm Syndrome (CSS) that needs to be managed by anti-cytokine therapy. Such therapies have been put forward for SARS-CoV-2 [48] and EBOV [49]. There are several similarities in the innate immune response generated upon SARS-CoV-2 and EBOV infection, which are illustrated in Figure 1.

Evasion of innate immunity has been proposed to be an immunological mechanism generally used by RNA viruses. Inhibition of type I interferon (IFN) response is identified to be primarily involved in this phenomenon, in SARS-CoV-2 [50] as well as EBOV. Mutations in genes involved in the regulation of type I and III IFN immunity leading to production of autoantibodies have been found to be linked with life threatening conditions in COVID-19 [51]. Moreover, COVID-19 has been found to become critical in individuals with inborn errors of type I IFN immunity [51]. In case of EBOV, type I IFN signaling is known to be inhibited by the Interferon inhibiting domain (IID) of the viral proteins VP35 and VP24 [52]. Interestingly, knockout mice deficient in interferon receptors are being used as experimental murine models for EVD, which underlines the involvement of interferon signaling in susceptibility to Ebola infection [53,54].

Neutrophil extracellular traps (NETs), originally described as a potential bacterial killing mechanism, is reported to be involved in the pathogenesis of viral diseases too [55]. NETosis, a regulated cell death mediated by neutrophils can follow the hyperinflammatory response to SARS-CoV-2 infection. Severe infection with SARS-CoV-2 results in release of inflammatory cytokines and chemokines which leads to release of NET, ultimately causing the clearance of the virus [56]. A multi-omics analysis of peripheral blood from EVD survivors also suggests involvement of NET associated proteins in causing tissue damage [57].

Inflammation is one of the major host defense mechanisms against viral infection. The lipid mediator, Prostaglandin E2 is known to play an important role in the associated inflammatory and immune responses [58]. In severe COVID-19, Prostaglandin E2 is shown to mediate impaired immune response [59]. Prostaglandin E is also demonstrated to be upregulated in Filoviral infection in bats [60], pointing towards the significance of prostaglandins in pathogenesis of EVD.

A life-threatening complication associated with both COVID-19 and EVD is disseminated intravascular coagulation (DIC), characterized by activation of coagulation system, leading to generation of microthrombi [61]. The infection of monocytes and macrophages with the virus results in release of tissue factor (TF), which is found to be important in the development of coagulopathy associated with COVID-19 [62] and EVD [63].

### 3.2. Alterations in Adaptive Immune Response

Adaptive immunity has two elements: humoral immunity and cell-mediated immunity, mediated by B-cells and T-cells, respectively. Once the virus enters the cell, activation of naive B cells leads to antibody production, mainly IgM followed by class switching to IgG. At the same time, activation of T-cells leads to further activation of CD4+ (Helper T cells) and CD8+ (Cytotoxic T cells). The cytokines secreted by the CD4+ T-cells facilitate the production of antibodies from B cells. The CD8+ T-cells on the other hand, help in the killing of infected cells. Upon encounter with the cognate antigen, some of B cells enter the germinal center and activate plasma cells leading to specific antibody production, with the help of T cells.

Sette et al. [64] propose that in the case of SARS-CoV-2 infections, the innate immune response is the first line of defense, as has been previously reported for any viral infection, and the adaptive response takes over soon after the waning of the innate response. The contributions of the innate and adaptive immunity become deeper with severity of the infection. 

#### 3.2.1. Overview of the Humoral Immune Response

**Figure 2:**
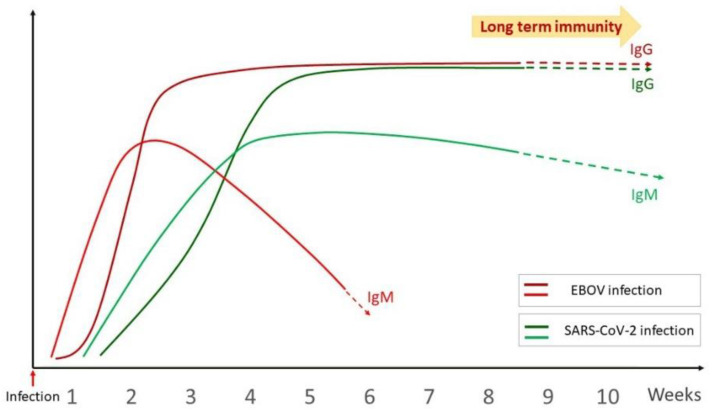
Comparative antibody response curve upon SARS-CoV-2 and EBOV infection.

The humoral immune response in viral infection is characterized by the secretion of neutralizing antibodies that protect the host against viruses. The humoral immune response to SARS-CoV-2 has been well studied. In one of the initial studies carried out by Long et al., 100% of patients exhibited anti-viral IgG within 19 days of infection [65]. IgG and IgM antibody titers have been found to be increased during the first 3 weeks after the onset of symptoms [65]. Similar studies have been carried out in EVD, with various patterns of observations. EVD specific IgG and IgM antibodies were found to be very low in patients who died of infection [66]. Analysis of EBOV specific IgG and IgM in 2014–16 Sierra Leone outbreak survivors indicated that humoral response develops in the first week of onset of symptoms and the concentration of antibody increases at the end of the second week of illness. IgG was detected earlier than IgM and the viral load was found to be negatively correlated with antibody titer [66]. IgM response preceding that of IgG response as well as both responses occurring at the same time have been demonstrated in different EVD patients in yet another study [67]. A comparative antibody response curve upon SARS-CoV-2 and EBOV infection has been illustrated in Figure 2.

Neutralizing antibodies, which play a significant role in viral clearance, are well correlated with long term anti-viral immunity [68]. Potent neutralizing glycan cap-specific monoclonal antibodies effective against multiple Ebola viruses have been demonstrated in survivors of EVD [69]. Wang et al. has studied the level of neutralizing antibodies in COVID-19 inpatients and convalescent patients and demonstrated its association with age, severity of disease and time after onset of symptoms [70].

Non-neutralizing antibodies are known to play a significant role in humoral immune response. In filoviral infection, non-neutralizing antibodies are also reported to confer protection that is mediated through the Fc domain [71]. This is achieved by antibody-dependent cellular cytotoxicity (ADCC) by natural killer (NK) cells or antibody-dependent cellular phagocytosis (ADCP) of viral particles or infected cells by Fc receptor-bearing cells [72]. A recent report indicates that the protection offered by non-neutralizing antibodies to SARS-CoV-2 are mediated by Fc-mediated effector functions [73] such as phagocytosis. 

There are reports that suggest a correlation between antibody kinetics and severity of COVID-19, with delayed antibody response observed in severe disease [74]. Characterization of antibody response to spike protein has revealed the existence of immunological imprinting, which refers to the ability of the immune system to recall existing memory cells developed in response to earlier betacoronavirus infections, rather than stimulating de novo responses [75].

Plasmablasts, known to be involved in formation of memory B-cells and plasma cells play a significant role in humoral immunological memory [76]. An increase in plasmablasts has been reported in COVID-19 [77,78] and EVD [67,79]. RBD specific memory B cell response to SARS-CoV-2 antigen is proved to evolve between 1.3 and 6.2 months after infection, in a study conducted by Gaebler et al. [80]. Assessment of immunological memory in SARS-CoV-2 revealed that the level of IgG against spike protein was stable over more than six months [81]. Presence of EBOV specific memory B cells has also been demonstrated in EVD survivors, which was found to increase with time post-convalescence [82]. 

Ever since the first recognized Ebola outbreak occurred in 1976, investigators have attempted to assess the long-term antibody kinetics in EVD survivors. Bebell et al. has reported that IgM was found to persist one to six months after infection whereas IgG persisted up to 10 years [83]. Reports also indicate the presence of antibodies specific to Ebola viruses 11 years post-infection [84,85]. Another study carried out 40 years after initial viral infection, in survivors of the first Ebola outbreak, interestingly revealed that 50% of the survivors displayed immunoreactivity to viral glycoprotein, nucleoprotein and VP 40, with the ability to neutralize live virus [86]. Similar attempts have been made in COVID-19 where long term kinetics studies reveal that neutralizing antibody responses could exist up to 13 months after SARS-CoV-2 infection [87]. More research is warranted on the antibody kinetics in long term survivors of various outbreaks caused by RNA viruses that could give more insights on long lasting viral immunity.

Adaken et al. carried out an interesting longitudinal follow up study of antibody levels in survivors of 2013–2016 Ebola outbreak which point towards a decay-stimulation-decay model. In this study, IgG levels are found to decrease following recovery, which then show a resurgence over a period of time [88]. Another 60 month observational prospective cohort study carried out with a larger sample size comprising 802 EVD survivors also evidenced a similar antibody ebb and flow pattern [89]. This periodic waxing and waning of antibodies observed in EVD, warrants investigation also in the context of COVID-19, since it may indicate sub-clinical de novo antigenic stimulation possibly indicative of viral RNA shed from immunologically privileged sites such as eyes, central nervous system and testes [90].

Difference in immunity with respect to gender has always been a matter of curiosity among the public and researchers. A study carried out in COVID-19 patients revealed a stronger antibody response in females compared to males, and this has been likely responsible for the lower fatality rates observed for females [91,92]. In contrast, there is no difference in the risk for SARS-CoV-2 infection between males and females, though severe forms are common among men [93]. It is worth noting that apart from biological reasons, women are more vulnerable to infections such as COVID-19 and EBOV due to occupational and domestic exposures [94].

#### 3.2.2. Landscapes in T-Cell-Mediated Immune Response

T-cells are major contributors to the adaptive immune response, playing diverse roles such as directly killing infected host cells, activation of other immune cells, production of cytokines and regulation of the immune response. T-cell response is very significant in deciding the course and clinical outcome of viral infections. CD4^+^ and CD8^+^ T cell subsets participate in T-cell immunity during viral infection; alterations of these two cell subsets have been widely studied in COVID-19 and EVD.

**Figure 3:**
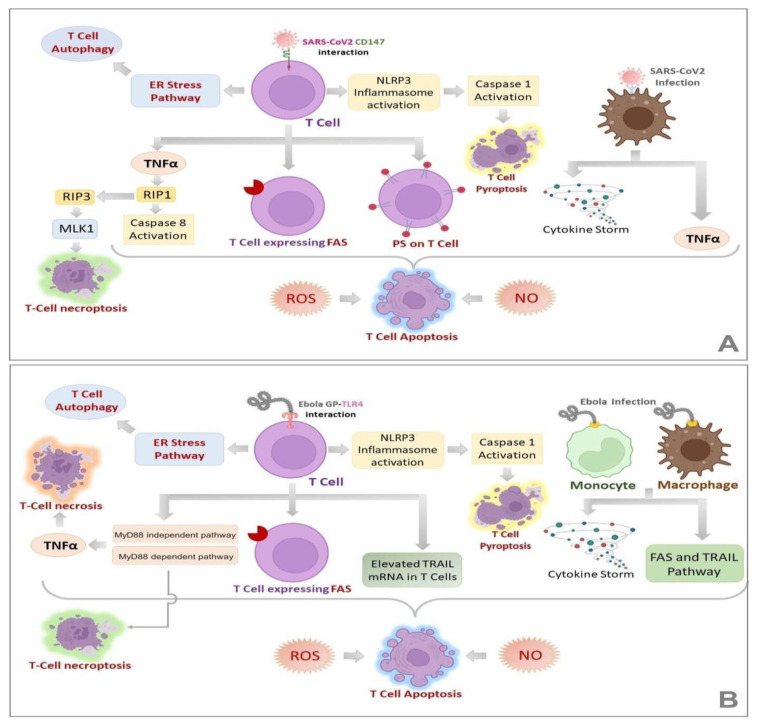
Mechanisms of Lymphopenia in SARS-CoV-2 (**A**) and EBOV (**B**) infections. Lymphopenia, a common feature of COVID-19 and EVD, is characterized by different routes of T-cell death such as apoptosis, necrosis, necroptosis and autophagy. (**A**,**B**) demonstrate the known mechanisms involved in each of these pathways in COVID-19 and EVD, respectively. It is noteworthy that many of these alterations are common for both the diseases, the fact that can be exploited for the design of novel pan-viral therapies.

**Figure 4:**
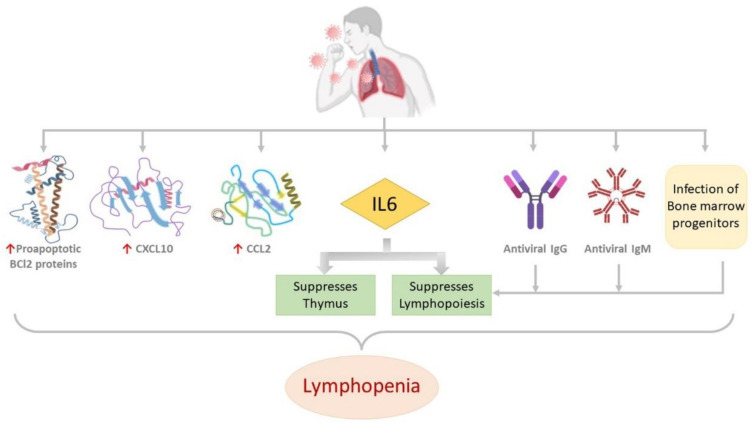
Molecular alterations associated with lymphopenia in COVID-19 patients. Proapoptotic BCl2 proteins, CXCL10, CCL2 and IL6 are upregulated. Antiviral antibodies IgG and IgM, infection of bone marrow progenitors and the upregulated IL6 suppresses lymphopoiesis. IL6 also suppresses the thymus. All these are reported to be associated with lymphopenia in COVID-19 patients.

Transient depletion of both CD4^+^ and CD8^+^ T-cells has been reported in COVID-19 infection [95] and EVD [96]; however, peripheral T-cell counts are preserved in asymptomatic COVID-19 patients [97]. Studies have found lymphopenia to be characteristically associated with critical COVID-19 [98], for which several theories have been put forward by researchers. Andre et al. have postulated T-cell apoptosis to be a major reason for T-cell depletion [99]. EVD is also characterized by deficiency in T-cell responses, apoptosis of lymphocytes and lymphopenia [52]. In vitro studies have demonstrated that EBOV binds to CD4^+^ T cells which is mediated by the interaction of GP with Toll-like receptor 4 (TLR4). This results in consequent production of Tumor necrosis factor α (TNF α) thereby stimulating apoptosis and necrosis of T lymphocytes [100]. Figure 3A,B illustrate various mechanisms that could lead to lymphopenia in SARS-CoV-2 and EBOV infections, respectively. Quite a few molecular alterations are reported to be associated with lymphopenia in COVID-19 patients, which are illustrated in Figure 4.

**Figure 5:**
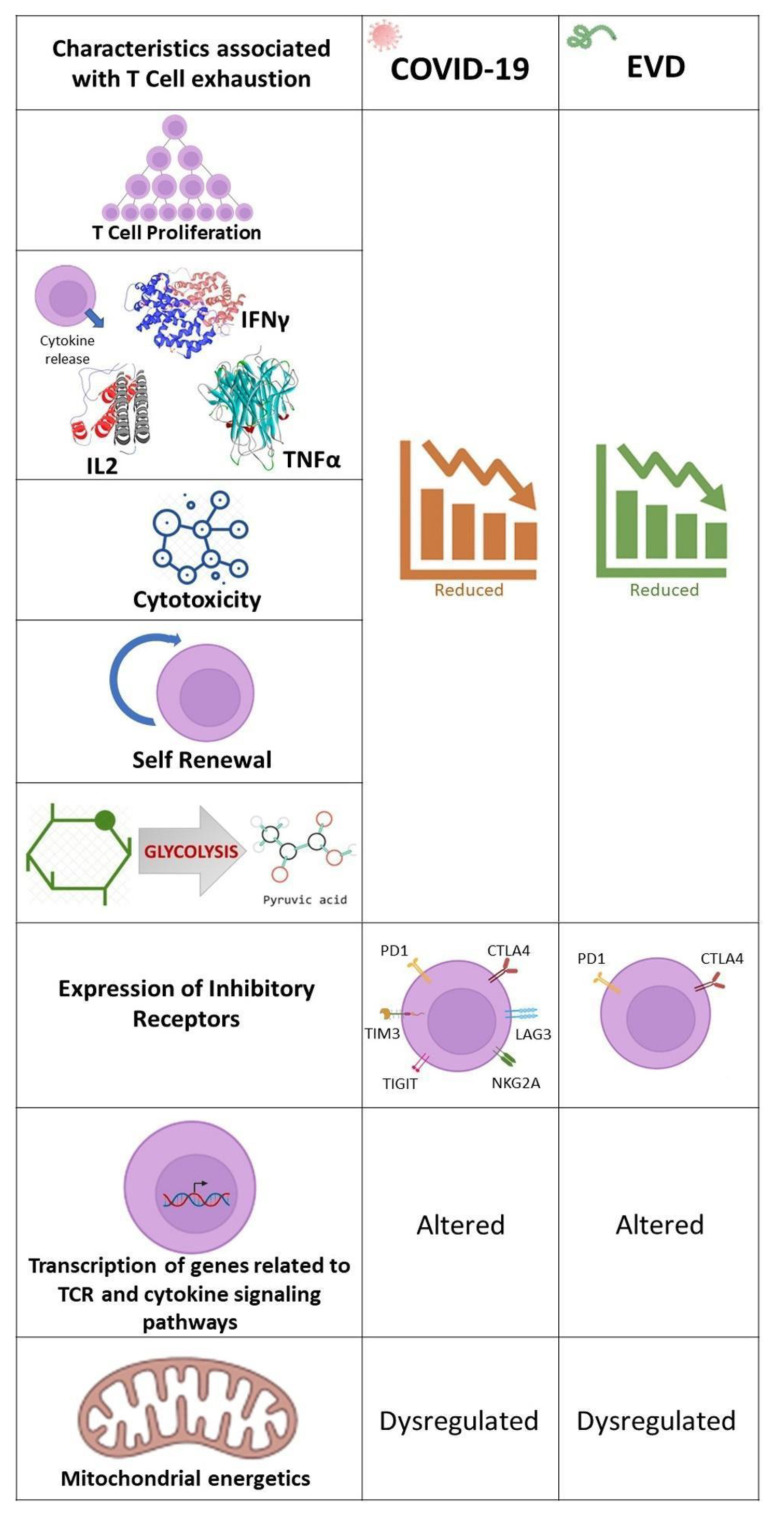
Factors associated with T-cell exhaustion in COVID-19 and EVD. Proliferation of T-cells, release of cytokines, cytotoxic and self-renewal capabilities of T-cells and glycolysis are commonly found to be reduced in COVID-19 and EVD. Additionally, T-cells express exhaustion markers, of which PD-1 and CTLA4 are common. Alteration of transcription of genes related to TCR and cytokine signaling pathways and dysregulation of mitochondrial energetics are also a common feature of T-cell exhaustion in COVID-19 and EVD.

T-cell exhaustion, which is a dysfunction or physical elimination of antigen specific T-cells [101] is a characteristic of several viral infections and has been reported in COVID-19 [102,103] and EVD [104]. The overexpression of Programmed cell death-1 (PD-1) and Cytotoxic T-lymphocyte-Associated protein 4 (CTLA-4), co-inhibitory receptors [105] is a common feature of T-cell exhaustion found in EBOV [106] and SARS-CoV-2 [107] infections. The percentage of CD4^+^ and CD8^+^ T cells expressing PD-1 and CTLA-4 has been found to be elevated in fatal EVD cases and reduced in survivors [106]. Additional inhibitory receptors such as Natural Killer group 2 member A (NKG2A) [108], TIM-3 [109], T cell immunoglobulin and ITIM domain (TIGIT) [110] and Lymphocyte-activation gene 3 (LAG-3) [111] have been hypothesized to be responsible for T-cell exhaustion in COVID-19. The various factors associated with T-cell exhaustion encountered in COVID-19 and EVD are illustrated in Figure 5.

#### 3.2.3. Long Term Anti-Viral Immunity

Two major concerns related to the long-term management of viral infections are recurrence of symptoms and reinfection. The persistence of virus at immunologically privileged body sites has been reported to cause recurrence of symptoms, after recovery, in COVID-19 and EVD patients. Investigators have detected EBOV in the urine, semen [112], conjunctiva, sweat, vaginal and rectal fluids [113], aqueous humor [114], breast milk and CSF [115] of recovered patients [116]. In addition, EVD survivors have reported contracting conditions such as uveitis [114] and neurological symptoms post-recovery [115]. The presence of SARS-CoV-2 virus in various body fluids has also been extensively studied and reviewed by some investigators [117,118].

In view of these facts, the survivors of EVD and COVID-19 may also pose a threat of transmission of the virus and cause new outbreaks. Such a resurgence of EBOV has been proven by the genome analysis of patients seven years after the viral outbreak in Guinea [119]. Evidences also indicate sexual transmission of EBOV from survivor to partner, several days after recovery [120,121,122]. The possibility of re-emergence of COVID-19 needs to be explored in this direction.

Reinfection by viruses depends on the immunity of recovered patients which may vary from person to person, resulting in variability of individual host susceptibility. Molecular phylogeny analysis of human infecting coronaviruses points to a probability of reinfection with SARS-CoV-2 between 3 months and 5.1 years after peak antibody response [123]. An evaluation of reinfection rates of COVID-19 reveals that only as little as 1% of the people who were previously infected, reported with reinfection [124]. However, the reinfection rate has rocketed to 10% since the emergence of Omicron, which supports the popular belief that Omicron is able to evade the immune responses induced by vaccination or previous infection [125]. In the case of EVD, confirmed reinfection cases in humans have not yet been reported though it is theoretically feasible [126]. However, reinfection has been demonstrated in mice and non-human primates with partial immunity [127,128].

Long-lived self-renewing T-cell memory is a fundamental property that results in swift and vigorous adaptive immunity on re-exposure to the same pathogen [129]. Both CD4^+^ and CD8^+^ are found to contribute to EBOV Glycoprotein (GP) specific T cell memory [130]. SARS-CoV-2 specific memory T cells have been found to be preserved in infected individuals irrespective of the severity of the disease [131]. Heterologous immunity, where memory T-cells generated upon encounter with a pathogen providing immunity against other novel pathogens has also been recently reported in SARS-CoV-2 patients, which could lead to varying severity outcomes [132]. This has been evidenced by the identification of pre-existing non-spike memory T-cells in SARS-CoV-2-naive contacts that offered them protection against COVID-19 [133]. A very striking finding is the production of virus specific T memory stem cells which are unique subcategories of memory T cells with self-renewing and multipotent abilities [134]. The presence of stem cells such as memory T cells has also been documented in both EVD [130,135] and SARS-CoV2 [131] convalescents.

The repercussions of emerging viral infections in survivors are a matter of great concern. EVD survivors are found to suffer from various illnesses including impotence, musculoskeletal pain, bleeding, ocular diseases, hearing loss, psychological problems which encompasses Post Ebola virus disease syndrome (PEVD) [136,137]. Similarly, post-acute COVID-19 syndrome characterized by persistent symptoms and long term complications such as dyspnea, fatigue, muscular weakness, joint pain, thromboembolism and anxiety are reported to occur from 4 weeks post-onset of symptoms [138]. Autoantibodies developed against self-antigens post-viral infection are documented to be associated with the above mentioned post-viral syndromes [139,140,141].

### 3.3. Risk Factors That May Influence the Immune Mechanism

Several host factors such as genetic susceptibility, age and sex, co-morbidity and immune compromised states are found to influence the immune responsiveness and hence the outcome of the viral infection. Right from the initial days of the pandemic, presence of co-morbid conditions such as chronic pulmonary obstruction, obesity, and hypertension have been linked to the severity of COVID-19 [142]. The compromised immune response is thought to be responsible for the very high increase in mortality and severity of COVID-19 seen among diabetics [143]. Moreover, disease-modifying therapies (DMTs) used for the management of comorbid conditions in COVID-19 have been shown to diminish the immune response [144,145]. Age has also been well studied as an immunological determinant of COVID-19 disease severity [146].

In the case of EVD, age and sex are not found to have any association with the risk of getting infected [147]. However, studies on the Sierra Leone outbreak indicate that the incidence rate increased with age and the median age of confirmed EVD cases was 28. Additionally, half of the infected cases were females [148]. As for coinfection, there is a dispute over association of *Plasmodium* species parasitemia with the prospects of surviving EBOV infection [149]. Such findings have been discussed in a systematic review by Edwards et al. [150]. Studies carried out in murine models warrant further research to prove the effect of *Plasmodium* coinfection on survival from EVD [151].

## 4. Impact of COVID-19 and EVD on Pregnancy

Alterations in hormone levels and the immune system in pregnancy may predispose pregnant women to viral infections. There have been many reports that indicate that viral infection during pregnancy is correlated with undesirable obstetric consequences [152,153,154]. In any viral outbreak, pregnant women constitute a high-risk group because of various reasons such as increased susceptibility and infrequent antenatal hospital visits due to fear of contracting the disease. Since they pose a risk for nosocomial infection to healthcare workers through exposure to body fluids, proper obstetric care might not be available to pregnant women. Various concerns regarding occurrence of viral infections in pregnancy are discussed below, focusing on COVID-19 and EVD.

Pregnancy is not believed to put women at higher risk of getting infected by EBOV [155,156] or COVID-19 [157]. However, in the case of COVID-19, there are studies that report a higher infection rate among pregnant women in comparison with non-pregnant women [158,159]. The high levels of estrogen and progesterone are thought to induce the upper respiratory tract to swell, which in turn could make pregnant women more susceptible to SARS-CoV-2 infection [160].

### 4.1. Maternal and Neonatal Outcomes Associated with COVID-19 and EVD

Several investigators have assessed the clinical complications and risk posed by SARS-CoV-2 and EBOV infections in pregnancy, with results of heterogeneous nature. Numerous aspects regarding maternal and neonatal outcomes in the context of COVID-19 and EVD need to be discussed so as to allow effective management of obstetric healthcare during viral outbreaks. Case studies and cohort studies relating to pregnancy and neonatal outcomes in COVID-19 and EVD have been summarized in Table 2a,b, respectively.

### 4.2. Effects of COVID-19 and EVD on Placenta and Vertical Transmission

The importance of the placenta in mediating inflammatory and immune responses has been profusely studied in pregnant women infected with SARS-CoV-2. A study of the placenta conducted in COVID-19 patients revealed that the placenta of infected women are characterized by conditions such as maternal thrombosis [191]. SARS-CoV-2 placentitis has been reported to be a complication of maternal COVID-19 infection and is likely to result in fetal compromise [192,193,194]. It has been reported that activation of robust placental immune response during maternal SARS-CoV-2 infection may contribute to poor pregnancy outcomes associated with COVID-19 and infection at the maternal-fetal interface is not even required for this [195]. Interestingly, fetal sex has been proposed to play a significant role in the maternal-placental-fetal immune crosstalk in the setting of maternal SARS-CoV-2 infection. Sexually dimorphic placental immune responses have been observed between male and female fetuses with associated reduced antibody transfer to male fetuses [196]. Still birth, macerated fetus, pre-term birth and miscarriages imply a role of placenta in adverse pregnancy outcomes in EBOV-infected women (Table 2). However, we could not come across any supporting studies in existing scientific literature and hence we suggest more investigations in this direction.

Vertical transmission of a pathogen can occur via placenta, vagina or breast milk [178]. Viruses in amniotic fluid and placenta are infectious and contribute to intrauterine transmission when they cross the placental barrier and infect the fetus [83,186]. This has been supported by the detection of SARS-CoV-2 virus in the placenta of COVID-19 positive women [197]. Additionally, among EBOV positive pregnant women, placenta has been reported to be a reservoir of the virus which could result in transplacental transmission to the fetus [178]. The mechanisms of macropinocytosis and clathrin-mediated endocytosis used by placenta for acquiring nutrients is exploited by EBOV to gain entry into the cells [198].

The placenta has specialized epithelial cells known as trophoblast cells that immunologically protect the neonate by controlling the spread of contagions [199]. Immunohistochemical analysis has evidenced the presence of Ebola viral antigens in syncytiotrophoblast, cytotrophoblast and placental maternal mononuclear cells [178]. SARS-CoV-2 is speculated to enter trophoblasts with the help of entry factors such as ACE2 and Transmembrane protease serine 2 (TMPRSS2) expressed on the villous placental trophoblasts [200]. Transplacental transmission of SARS-CoV-2 has been documented in neonates born to mothers infected in the last trimester [201]. Case studies carried out in EBOV-infected pregnant women whose fetus died in utero also suggest infection of the fetus through the placenta [182,186].

Vertical transmission potential of SARS-CoV-2 has been discussed in the earlier days of the pandemic itself, when a neonate born to a COVID-19 positive mother had elevated IgM antibody levels [202]. Fenizia et al. reported around 10% vertical transmission in SARS-CoV-2-infected mothers [203]. Incidentally, maternal SARS-CoV-2 infection in the third trimester has been reported to cause compromised placental antibody transfer, possibly due to compensatory mechanisms that boost immunity in the neonates [204]. However, some authors think that the risk of vertical transmission of SARS-CoV-2 to neonates is very low [205,206]. To our limited knowledge, there are no studies illustrating transplacental antibody transfer with regard to EBOV infection.

There are several concerns regarding breastfeeding in the context of viral diseases, where the risk for the infant contracting the disease through breast milk remains a research question. In the case of SARS-CoV-2, reports indicate that viral RNA is rarely found in breast milk [207] and even when present, it does not represent a risk factor for transmission to the infant [208]. However, investigators have detected EBOV in breast milk [209,210,211], and the viral RNA was found to persist in breast milk of EBOV positive mothers up to 500 days post-treatment [212]. A systematic study demonstrated that 80% of children who ingested EBOV positive breast milk died of the disease [209]. This indicates the risk of mother to child transmission of EBOV through breast milk, though the inadequate number of samples studied is insufficient to emphasize it. Moreover, WHO guidelines recommend discontinuing breastfeeding when a woman is suspected of EBOV infection [156].

The presence of EBOV in female genital tracts in animal models [213] and vaginal fluid in infected humans [214] suggests the possibility of transvaginal transmission of the virus from mother to neonates. The presence of SARS-CoV-2 virus has also been detected in the lower genital tract of women with COVID-19 infection [215]. On the other hand, certain investigators have reported that SARS-CoV-2 virus is rarely detected in vaginal secretions [216,217].

The impact of mode of delivery on pregnancy outcomes and intrapartum transmission of the virus has been debated at large. A systematic review conducted by Cai et al. concluded that there is no significant difference in the rate of neonatal infection, neonatal deaths and maternal deaths between women who had undergone vaginal and cesarean delivery [218]. However, certain other studies have demonstrated association of vaginal delivery with a low risk of intrapartum transmission of SARS-CoV-2 [219,220]. In another cohort study, women who underwent cesarean section were reported to face adverse maternal and neonatal outcomes, while women with mild symptoms who delivered vaginally had excellent outcomes [221]. Hence, it can be inferred that cesarean delivery is no better than vaginal delivery in preventing adverse outcomes and the decision for the mode of delivery may be based on individual cases and the severity of the disease.

### 4.3. Alterations in Immune Response in Pregnant Women with SARS-CoV-2 and EBOV Infections

**Figure 6:**
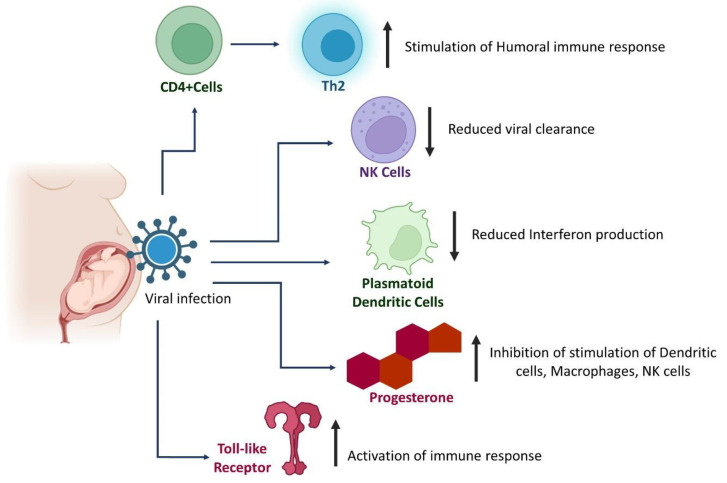
Alterations in immune response in pregnant women during viral infections.

Pregnancy is a unique immunological condition that attracts intense discussion on the need to evaluate the risk posed by the exposure of pathogens to the materno-fetal unit [222]. During pregnancy, the mother has to adapt to the semi-allogenic fetus and at the same time sustain defense against pathogen attack. A proinflammatory atmosphere prevails during the first trimester while an anti-inflammatory environment builds up during the second and third trimesters to assist fetal growth [223]. Pregnant women exposed to viral infections experience several significant alterations in immune response which are summarized in Figure 6.

COVID-19 infection in pregnancy is accompanied by higher levels of neutrophils and C-reactive protein that indicate an enhanced innate immune response, suppressed cytokine storm and increased activity of CD8+ T-cells and NK cells [224]. Neutrophil activation has also been reported in neonates born to women infected with SARS-CoV-2 [225]. IL-8, a pro-inflammatory cytokine that plays a key role in neutrophil recruitment, has been found to be increased in SARS-CoV-2-infected mothers and their neonates [226]. Pregnant women with moderate or severe COVID-19 were also observed more likely to have leukopenia [227]. Alterations in innate and adaptive immune response are present at the maternal-fetal interface as evidenced by studies carried out in decidua basalis tissues collected at the time of delivery from women with COVID-19 infection, and these changes are found to be correlated with the gestational stage of maternal infection [228].

The innumerable pregnancy complications and fetal outcomes reported in women infected with EBOV, accompanied by very high maternal and fetal mortality rates, as discussed in Table 2, indicate a possibility of an adverse immune response in pregnant women with EVD. Nevertheless, we could hardly find any literature investigating the immune response in EBOV-infected pregnant women. More research is required to answer queries regarding cellular sources and targets of inflammatory molecules in placenta so as to develop approaches to circumvent the obstetric intricacies in EVD.

## 5. Immunomodulatory Approaches for Combating COVID-19 and EVD

A lack of effective therapeutic agents against emerging viral diseases such as COVID-19 and EVD makes it difficult to manage the symptoms and outcomes of these ailments. Since there is a marked alteration in the immune response in these infections, immunomodulatory agents should be further explored for their therapeutic and prophylactic potential. Immunomodulatory agents fall into two categories depending on their mechanism of modulation, namely, immunostimulants and immunosuppressants [229]. Diverse immunomodulatory approaches currently employed for combating COVID-19 and EVD have been shortlisted in the Supplementary Table S1.

### 5.1. Vaccines against SARS-CoV-2 and EBOV

Vaccines belong to the class of specific immunostimulants that generate an immune response against a particular pathogen. The pandemic speed at which COVID-19 vaccines were developed can be hailed as a technological breakthrough [230,231]. Presently, 38 COVID-19 vaccines are approved for use across the world and they have been developed using various platforms [232]. In the case of EBOV, around thirteen vaccines have been subjected to human trials, out of which two vaccines are approved [233]. Table 3 lists the immune responses, challenges and adverse events related to approved vaccines developed against COVID-19 and EVD. Studies of the immune alterations in response to vaccination are necessary to plan successful and long-lasting immunization strategies.

Clinical trials are required to assess the safety and efficacy of vaccines before they are applied for mass immunization. Several clinical trials of vaccines against COVID-19 and EVD are currently going on across the globe; the status and other details of clinical trials of vaccines employing common platforms are summarized in Supplementary Table S2. Booster doses are warranted to offer complete protection from the disease, particularly when the immunity provided by the primary vaccination starts to wane off or when the original vaccines no longer act against the antigens presented by the new variants. Research output on COVID-19 demonstrates that improvement in immunological response to vaccine antigen and reduction in the risk of infections are well associated with boosting [265]. The benefits of booster dose have also been demonstrated in EVD where the bivalent Ebola virus-like particle (VLP) vaccine is reported to cause clonal increase of GP specific T cells which was found to elevate after the second booster dose [266]. The strategy of prime-boost immunization is found to offer maximum protection against EBOV infection both in case of Zabdeno [267] and cAd3-EBOZ [268] vaccines, where boosting is done by administration of modified vaccinia Ankara (MVA) strain [233]. Similarly, heterologous boosting has been reported to offer substantial protection against mild and severe COVID-19 [269]. 

In the case of COVID-19, a fourth dose of vaccine has been offered in Israel to its population above 60 years and health care workers [270], though there are reports that the fourth dose of mRNA vaccine does not offer any additional protection against new variants such as Omicron [271]. Ultimately, boosters are helpful from the perspective of the general population, but adverse immune mediated side effects should be weighed properly before widely introducing the booster doses [251]. This is particularly true in the case of immunocompromised individuals, among whom an investigation has demonstrated lower effectiveness of mRNA vaccination, in comparison to immunocompetent individuals [272].

Another aspect that needs to be discussed is the immune response that is mounted after a natural infection in comparison with that achieved after vaccination. The neutralizing antibody response generated by all the approved COVID-19 vaccines is similar to or higher than that generated in convalescent individuals [273]. A comparative study of longitudinal dynamics of the immune responses has revealed that three months post-vaccination, the cellular immunity was comparable while the humoral response was stronger in vaccinated individuals compared to COVID-19 recovered patients [274]. However, the antibody titers were found to decline 1 to 3 months post-vaccination [275]. In the case of EVD, a cohort study evaluating vaccination and EBOV infection has observed more efficient immunity in survivors than vaccinated volunteers. Higher neutralizing capacity, larger pool of neutralizing antibodies and elevated NK cell activation were observed in the survivors [276].

We would like to touch upon another interesting concept with respect to immune response in vaccination of COVID-19 recovered patients. Sariol et al. has demonstrated that antibodies generated after natural infection, though similar in quantity, are better in their function, when the natural infection preceded vaccination [277]. Such observations reinforce the concept of hybrid immunity that is proposed to develop in vaccinated individuals who recovered from COVID-19. The antibodies that develop in such people post-vaccination, have been reported to neutralize immune evading strains such as the Beta variant, when compared to vaccinated individuals who have never been infected [278]. Infection-acquired-immunity, when boosted with vaccination, is also found to last longer [279]. A similar study has been published from India, in which the COVID-19 recovered individuals who received just a single dose of Covishield were found to mount a much higher immune response in comparison with those who have been vaccinated with two doses [280]. Additionally, individuals with hybrid immunity are reported to require much lower hospitalization associated with SARS-CoV-2 reinfection [281]. All these data were analyzed prior to the emergence of Omicron, and hence experts warn that the relevance of these results need to be reassessed in the current scenario, after the advent of new variants including Omicron [282].

Patients with comorbid conditions are always assessed to be at greater risk and hence are universally prioritized for vaccination. Anti-CD20 therapy such as Rituximab, which is considered a significant management strategy in auto-immune diseases and cancer, is found to reduce the immune response to vaccination [283] and hence adequate interval between the Rituximab treatment and COVID-19 vaccination has been proposed [284], [285]. Furer et al. have carried out a multicentric study that demonstrated BNT162b2mRNA vaccine to be immunogenic in patients with autoimmune inflammatory rheumatic diseases. However, anti-CD20 therapy in these patients resulted in a substantial reduction of vaccine induced humoral response [286]. Assessment of safety and efficacy of EVD vaccines in patients with comorbidities is scarce in literature and hence warranted in view of the high mortality rate of the disease.

### 5.2. Vaccination in Pregnant Women against COVID-19 and EVD

Pregnant women are normally excluded from the initial clinical trials of vaccination against any infectious disease due to various apprehensions among the public concerning safety. This scenario has led to the generation of very little research data regarding the safety and efficacy of vaccination in pregnant women against COVID-19 [287] and EVD [288] until now. The scarce and inconsistent literature is one of the reasons for the still-persisting vaccine hesitancy among pregnant women [289]. However, the emerging reports from various corners of the globe are promising and need to be exploited for educating the public and spreading awareness on vaccination. We have included the available data regarding the immune response and outcomes of vaccination in pregnant women against both COVID-19 and EVD in Table 3.

## 6. Conclusions

The greatest threat to humanity in the present day is the emergence and re-emergence of highly pathogenic RNA viruses that cause diseases such as Influenza, Zika virus disease, Encephalitis and Dengue fever. Researchers could help in the development of therapeutic and prophylactic strategies for effective management of COVID-19 in such a short span of time only because of the foundations laid by innumerable studies carried out previously on various viral diseases. Since COVID-19 was a pandemic outbreak, scientific investigators around the world had immediately focused on generating research outputs needed to combat the disease. Though COVID-19 research was all-encompassing, many perspectives had not been addressed in diseases such as EVD probably due to the non-availability of sufficient samples because of higher mortality. More studies are required to unravel the latent aspects in the immunology of EBOV infection in pregnant and vaccinated individuals, which could aid in designing novel therapeutic approaches. We hope that the present review kindles rigorous research that may yield promising findings that could be extrapolated to other RNA viruses.

## Figures and Tables

**Table 1:** Comparison between the structural aspects of EBOV and SARS-CoV-2 pathogens.

	SARS-CoV-2	Ebola
Virus family	Coronaviridae	Filoviridae
Genome	+ssRNA~30kb [23]	−ssRNA~19 kb [24,25]
Structural proteins and their names	4 Proteins-Spike (S), Membrane (M), Envelope (E) and Nucleocapsid (N)	7 proteins-nucleoprotein (NP), polymerase cofactor (VP35), matrix protein (VP40), minor matrix protein (VP24), glycoprotein (GP), transcription activator (VP30) and RNA-dependent RNA polymerase (L) [24,25]
Non-structural proteins and their names	16 Proteins-Nsp1-16 [26,27]	2 Proteins-soluble GP (sGP), small soluble GP (ssGP) [28]
Viral Surface Protein mediating entry	S-Protein [15]	Glycoprotein (GP) [25,29]
Target cells	Macrophage, monocytes, lymphocytes, platelets cardiomyocytes, bronchi, trachea, Pneumocytes, respiratory ciliated cells, alveolar cells, intestinal epithelial cells [20,23]	Monocytes, macrophages, dendritic cells, hepatocytes, adrenal cortical cells and endothelial cells [22,25,30,31,32]
Target host cell receptor	ACE2 BSG/CD147 CD209L/L-SIGN CD209/D-SIGN Siglec-1, [20,23,30,33,34]	TIM-1 [21,22]
Incubation Period	2–14 days [35,36]	2–21 days [25]
Mode of Transmission	Aerosols, respiratory droplet [5]	Body fluid [24]
Nature of outbreak	Pandemic	Epidemic

ACE2—Angiotensin-Converting Enzyme-2; Nsp—non-structural proteins; −ssRNA—Single negative sense RNA; +ssRNA-Single positive sense RNA; TK—tyrosine kinase family members; TIM-1—T-cell immunoglobulin mucin domain 1; NPC1—Niemann–Pick disease type C1 protein; DC-SIGN—DC-specific ICAM-3-grabbing nonintegrin; Siglec-1/CD169—sialic acid-binding Ig-like lectin 1; BSG-cell surface protein Basignin.

**Table 2:** (**a**) Case studies and cohort studies relating to pregnancy and neonatal outcomes in COVID-19. (**b**) Case studies and cohort studies relating to pregnancy and neonatal outcomes in EVD ETU-Ebola Treatment Unit.

**(a)**													
**Year of Study**	**Country**	**Study Approach**	**Case Type**	**Sample Size**	**Pre-Existing Medical Conditions**			**Need for Hospitalization**		**Maternal Mortality**	**Obstetric Complication**	**Perinatal Outcome**	**Reference**
2020	22 countries	Retrospective cohort study	Pregnant women with confirmed SARS-CoV-2 infection	388	Not available			ICU admission (11.1%) Mechanical Ventilation (9.3%)		0.80%	Miscarriage (19.4%) Termination of pregnancy (1.1%)	Termination of pregnancy (1.1%) Pre-term birth (26.3%) Still birth (2.3%) Neonatal death (2%) SARS-CoV-2 positive (0.4%)	[161]
2020	United Kingdom	Prospective observational cohort study	pregnant women with confirmed SARS-CoV-2 infection	427	Asthma (7%) Hypertension (3%), Diabetes (3%)			Critical care (10%)		1%	Pregnancy loss (1%)	Stillbirth (1%) Neonatal death (1%) Loss of Pregnancy (1%) SARS-CoV-2 positive (2%)	[162]
2020	Singapore	Prospective Cohort Study	Pregnant women with diagnosis of COVID-19	16	Asthma (12.5%) HCV carriers (6.25%)			ICU admission (6.25%)		NIL	Spontaneous miscarriage (22.2%)	Neonatal death (6.25%)	[163]
2020	China	Retrospective Cohort study	Pregnant women who gave a single live birth between January 13 and March 18, 2020	65	Not available			Higher need for Caesarean section (80%)		NIL	Gestational diabetes (3%) Gestational hypertension (11%) Pre-eclampsia (1%)	Pre-term birth (14%) Diarrhea (1.7%) Fever (5.17%)	[164]
2020	Iran	Prospective Cohort Study	Pregnant women with diagnosis of COVID-19	56	Diabetes (16.1%) Hypertension (10.7%) Hypothyroidy (19.6%)			ICU admission (10.7%) Mechanical Ventilation (6.15%) Higher need for Caesarean section (67.3%)		NIL	Pre-eclampsia	Pre-term birth (34.5%) Perinatal death (3.6%)	[165]
2020	France	Retrospective Cohort study	Pregnant women with diagnosis of COVID-19 having a code for hospitalization for COVID-19	874	Diabetes (1.3%) Hypertension (1.9%)			ICU admission (5.9%) Higher need for Caesarean section (33%)		0.20%	Pre-eclampsia (4.8%) Gestational hypertension (2.3%) Postpartum hemorrhage (10%)	Pre-term birth (11.3%)	[166]
2020	Democratic Republic of the Congo	Case Study	Pregnant woman with confirmed SARS-CoV-2 infection	1	NIL			Caesarean section		NIL	Thrombotic vasculopathy in the placenta, Inflammatory appearance in the pelvic organs	SARS-CoV-2-infected Neonate, Perinatal death	[167]
2020	China	Retrospective Case Control study	Pregnant woman with confirmed SARS-CoV-2 infection, pregnant women with suspected infection and Control groups	11	Gestational diabetes (18.75%) Gestational hypertension (18.75%) Hypothyroidism (12.5%)			Caesarean section (87.5%)		NIL	Pre-eclampsia (6.25%)	Pre-term birth (18.8%), Low birth weight (17.6%)	[168]
2020	China	Case Study	Pregnant woman who was exposed to SARS-CoV-2	1	NIL			Hospitalization Caesarean section		NIL	NIL	SARS-CoV-2-infected Neonate	[169]
2020	USA	Case Series	Pregnant women with suspected COVID-19 infection	92	NIL			Hospitalization (1.1%)		NIL	low morbidity	One fetal demise, but not sure whether it is due to COVID-19	[170]
2020	Sweden	Case Series	Critically ill pregnant or newly delivered women positive for COVID-19	5	Gestational diabetes (2 out of 5) Gestational Hypothyroidism (1 out of 5) Situs Inversus (1 out of 5)			Hospitalization for an average of 20 days (4 out of 5) Intubation (4 out of 5)		NIL	Severe respiratory distress syndromeCardiac arrest (1 out of 5)	NIL	[171]
2020	USA	Retrospective cohort study	Possible exposure or infection and positive COVID-19 test	1609	Chronic pulmonary disease (12.6%) Cardiac arrhythmia (10.4%) Hypertension (6.5%) Hypothyroidism (5%) Diabetes (3%)			Hospitalization (60.5%)		0.20%	Not available	NIL	[172]
2020	USA	Retrospective cohort study	Pregnant and post-partum patients with SARS-CoV-2 infection	2352	Chronic pulmonary disease (12%) Hypertension (6.9%) Thyroid disease (3.9%) Diabetes (3.8%)			ICU admission (3.7%)		0.20%	Post-partum hemorrhage (2.6%) Other infections (2.3%) Hypertensive disorders of pregnancy (10.1%)	Fetal/neonatal death (2.5%) Miscarriage (1.2%) Stillbirth (0.5%) Preterm birth (17.7%)	[173]
2020	USA	Observational Cohort study	Women who delivered and had SARS-CoV-2 infection during pregnancy	252	Gestational diabetes (3%) Chronic hypertension (5%)			Hospitalization (6%)		NIL	Pre-eclampsia (11%) Chorioamnionitis (10%) Excessive blood loss (7%)	Neonatal SARS-CoV-2 infection (3%)	[174]
2020	Iran	Retrospective case Control study	Pregnant women with COVID-19 positive test and a positive chest X-ray result	110	Hypertension (5.45%) Diabetes (9.09%) Asthma (5.45%)			ICU admission (6.9%) Requirement for invasive ventilation (1.7%)		NIL	Abortion (21.42%) Post-partum hemorrhage (5%) Pre-term birth (25%)	Still birth (5%) Fetal distress (10%) Low birth weight (10%) NICU admission (10%)	[175]
2021	18 countries	Cohort study	Pregnant women with diagnosis of COVID-19	706	Hypertension (3.7%), Diabetes (4.7%), Chronic respiratory disease (3.5%), Endocrine dysfunction (10.6%)			ICU admission (8.4%)		1.60%	Hypertension Pre-eclampsia Anemia Infections	Pre-term birth (22.5%) Low birth weight (20.5%) SARS-CoV-2 positive (57.1%)	[176]
(**b**)													
**Year of Study**	**Country**	**Study Approach**	**Case Type**	**Sample Size**	**Maternal Age**	**Gestational Age of Infection**	**Comorbidity**	**Clinical Presentation**	**Need for Hospitalization/ICU Admission**	**Maternal Mortality**	**Obstetric Morbidity**	**Perinatal Outcome**	**Reference**
1995	Kikwit	Cohort Study	Ebola positive Pregnant women	15	24–38 (mean age 32)	First trimester (27%), second trimester (40%) and third trimester (33%)	Not available	Fever (100%), asthenia (100%), abdominal pain (100%), conjunctivitis (100%), anorexia (100%), diarrhea (100%), arthralgia (100%), dysphagia (100%), headache (100%)	Admitted to General Hospital	95.5% death	Genital bleeding (100%)	Abortion (67%), curettage performed due to incomplete abortion (20%), still birth (6.7%)	[177]
2000	North Uganda	Case study	Ebola positive Pregnant women	1	31	28 weeks	Placenta had a moderate amount of malarial parasite pigment	Conjunctival injection, diffuse abdominal tenderness, and slight pulmonary rales	Admitted to ETU	Maternal survival	Placenta had mild subchorionitis	Still birth	[178]
2012	Congo	Case study	Ebola positive Pregnant women	1	29	7 months	Not available	Fever, vomiting, dysphagia and diarrhea, drowsiness and wheezing, Dyspnea, coma stage 1b, light exophthalmos, cold limbs and sub icterus	Admitted to ETU	Maternal death	Dystocia	Death of neonate	[179]
2014	Liberia	Case Study	Ebola positive Pregnant women	1	31	Third trimester	Not available	vomiting, diarrhea, bleeding, and semi consciousness	Admitted to ETU	Maternal death	Not available	Intrauterine fetal death	[180]
2014	Guinea	Case Study	Ebola positive Pregnant woman	1	40	4th month	Not available	abdominal pain, diarrhea and fever	Admitted to ETU	Maternal survival	Vaginal bleeding	Still birth	[181]
2014	Southern Guinea	Case study	Ebola positive Pregnant women	2	20’s	7 months	Malaria (50%)	Asthenia, fever, and vomiting, Anasarca (50%)	Admitted to ETU	Maternal survival (100%)	Absence of uterine contraction, cervical dilation (50%) and fetal heartbeat, hypertonic uterus (50%), post-partum hemorrhage (50%), suspected chorioamnionitis (50%)	Still birth (100%)	[182]
2014	Sierra Leone	Case study	Ebola positive Pregnant women	1	34	36	Not available	Headache, cough, and arthralgia	Admitted to ETU	Maternal survival	Hydropic Placenta	Still birth	[183]
2014	Sierra Leone	Cohort Study	Ebola positive Pregnant women	55	Mean age 25	Not available	Not available	Fever (86.8%), fatigue or weakness (81.1%), nausea or vomiting (64.2%), headache (66%), muscle or joint pain (58.5%), vaginal bleeding (32.1%), unexplained bleeding (20.8%), and sore throat (13.2%)	Admitted to ETU	Not available	Vaginal bleeding (32%)	Not available	[184]
2014	Sierra Leone	Cohort Study	Ebola positive Pregnant women	67	Mean age 23	28–37 weeks	Not available	Fever (86.8%), abdominal pain (75.5%), fatigue (81.1%), nausea (64.2%)	Admitted to ETU	Maternal death (79%)	Vaginal bleeding (32%), obstetric hemorrhage (29.8%) and eclampsia (1.5%)	Spontaneous abortion (20.9%), Fetal death (5 out of 6), Still birth (8)	[185]
2014	Sierra Leone	Case study	qPCR negative, IgG positive	1	19	36 weeks	Sickle cell anemia	Symptom free	Admitted to ETU	Maternal survival	Not available	Intrauterine fetal death, heavily macerated baby	[186]
2014–15	Liberia and Sierra Leone	Retrospective Cohort study	Ebola positive Pregnant women	13	20-32	Not available	Not available	Abdominal pain (85%) and nausea/vomiting (69%), Bleeding (30%), Hiccups (8%) and non-hemorrhagic rash (8%)	Admitted to ETU	46% death	Not available	Preterm delivery (15%), Perinatal death (15%), Abortion (15%), Termination of pregnancy (7.6%),	[187]
2014–2015	Sierra Leone	Case series	Ebola positive Pregnant women (83.3%), Ebola survivor (16.6%)	6	18-38	Third trimester	Not available	Muscle pain (16.6%), headache (16.6%), diarrhea (16.6%), vomiting (16.6%)	Admitted to ETU	Maternal death (66.6%)	Postpartum hemorrhage (50%), hypovolemic shock (16.6%)	Neonate death (83.3%), still birth (16.6%)	[188]
2015	Sierra Leone	Case Study	Ebola positive Pregnant woman	1	22	5 months	Not available	Anorexia, muscle pain, and joint pain	Admitted to ETU	Maternal survival	Leaking fluid	Intrauterine fetal death	[181]
2015	Sierra Leone	Case Study	IgG, IgM positive	1	20		Not available	Severe back pain, loss of appetite, and intense fatigue	Delivery attended by village traditional birth attendant	Maternal survival	Leakage of bloody fluid from vagina	Still birth	[189]
2016	Guinea	Case Study	Ebola positive Pregnant women	1	25	28th week	Not available	Hyperthermia, asthenia, and conjunctival infection	Admitted to ETU	Maternal death post delivery	Severe vaginal bleeding with signs of coagulopathy	Survived after treatment	[190]

**Table 3:** Immune responses of few COVID-19 and EVD vaccines currently approved by various agencies.

Vaccine Platform	Name of Vaccine	Approving Agency	Immune Response	Efficacy on Original Variant % (95% CI) after Complete Regimen	Challenges	Adverse Event Following Immunization	Vaccination on Immunocompromised Patients (Immunogenicity)
COVID-19							
mRNA	Comirnaty (BNT162b2)	The Food and Drug Administration of the United States of America and Health Canada and The European Medicines Agency	S1-specific IgA and IgG Plasma nAbs [234]	95 [235,236]	LNP temperature sensitive no previous RNA based vaccine potential genetic risks since mRNA is unstable [237,238]	SAE + headache, fever, fatigue	Hemodialysis-reduced by the Uremic condition [239] Kidney TP-Strong Inhibition [239] Pregnancy—no difference of binding, neutralizing, CD4+ CD8+ T cells, IgG Ab to fetus via placental transfer, breast milk, no adverse outcomes, safe to administrate 3rd trimester [237,238,240,241]
	SpikeVax (mRNA-1273)	The European Medicines Agency and The Food and Drug Administration of the United States of America and Health Canada	Anti-S1 Abs [242]	94.1 [243]	LNP temperature sensitive potential genetic risks since mRNA is unstable [244,245]	SAE + myalgia, arthralgia, chills, fatigue, fever, axillary tenderness nausea/vomiting	Kidney TP-No immune response due to diabetes and antithymocytes globulins treatment [246] Hematologic malignancies-high rate of both humoral and cellular response [243] Pregnancy—no difference of binding, neutralizing, CD4+ CD8+ T cells, IgG Ab to fetus via placental transfer, breast milk, no adverse outcomes [237,238,240]
Adeno vector	VaxZervria/AZD1222/ Covishield (ChAdOx1_nCoV19)	Ministry of Food and Drug safety, Republic of Korea Central Drugs Standard Control Organization, India	Anti-RBD IgG, Anti-S1 IgG, nAbs [247]	70.4 [248]	Pre-existing immunity to the vector reduces vaccine effect [244,245]	SAE + Fever, headache, muscle ache, malaise, chills	Pregnancy—No increased risk of miscarriage, no instance of still birth, not affect fertility [152,249] Kidney TP-effectively induce humoral response [249]
	Janssen (Ad26.COV2. S)	The European Medicines Agency and The Food and Drug Administration of the United States of America and Health Canada	nAbs [250]	66.9–76.7 [236]	Risk of rare immune mediated thrombotic events and thrombocytopenia	SAE + blood clots, Muscle ache and nausea	Pregnancy—safe to administrate 3rd trimester, efficacy, safety, immunogenicity similar to general population [241]
	Sputnik/Gam-COVID-Vac (rAd26þrAd5)	The European Medicines Agency	ACE receptor blocking Abs, anti-RBD, Enhanced levels of IFNγ, CD107a expressing T cells, memory B cells, [251]	91.6 [236]	-	Flu-like illness, injection site reactions, headache, asthenia	Pregnancy—no effect on fertility, no adverse effect and complication [152]
Whole Inactivated Vaccine	CoronaVac	National Medical Products Administration (NMPA)	SARS-CoV-2 nAb [234]	83.5 [249]	Risk of vaccine enhanced disease Possible Th2-bias Hypersensitivity Live-attenuated vaccine against complex pathogens are challenging [244,245]	SAE+ allergic reaction, cough, myalgia, chill, nausea	Pregnant women-Effective in prevention, severe illness [252] HIV-Immunogenicity reduced compared to healthy [253] ARD-Safe, reduced, Short-term immunogenicity [254]
	CoVAXIN (BBV152)	Central Drugs Standard Control Organization, India	Enhanced plasma levels of CCL_4_, CXCL_1_, CXCL_2_, CX_3_CL_1_, IFNγ, IL-2, TNFα, IL-4, IL-5, IL-10, IL-13, IL-17A, IL-6,IL-12, IL-1α, IL-1β, IL-3, IL-7 Diminished plasma levels of CXCL_10_, IL-25, IL-33, GM-CSF and type 1 IFNs. [255]	77.8 [236,249]		SAE+ Fever, myalgia, Nausea, vomiting	Kidney TP-effectively induce humoral response [256] Pregnancy—Insufficient data
	Covilo (BBIBP-CorV)	Chinese National Regulatory Authority (NRA), National Medicinal Product Administration (NMPA)	No data available	78.1 [249]		SAE+ Fever, headache, cough	Breast cancer-anti-S-RBD ab, no interference with trastuzmab [257,258] Heart block-increased risk, may added side effects, humoral response interferes with conduction system of heart [259] Pregnancy—No data available
Recombinant protein	NVX-CoV2373 (Nuvaxoid)/Covovax	Central Drugs Standard Control Organization, India	Th1 response, anti-spike IgG, nAbs [260]	89.7 [236]	Availability of appropriate adjuvant Weak immunogenicity	SAE + erythema, tenderness, malaise, myalgia, nausea/vomiting	Pregnancy—No data available HIV-attenuated humoral response [261]
Ebola Virus Disease (EVD)							
Recombinant VSV	Ervebo (rVSVΔG-ZEBOV/, rVSVΔG-ZEBOV-GP)	The Food and Drug Administration of the United States of America and Health Canada and The European Medicines Agency	Increase in Total IgG, nAbs	94.5 [262]	Reports of arthritis in a subset of vaccinees Infectious virus found in synovial joints of vaccinees Interaction and interference between vaccine and therapeutics [233]	SAE+ Joint pain +rash+ Myalgia + arthritis + nausea + chill	Pregnancy—No significant & conclusive data available HIV—Safe, decreased immunogenicity [263]
Adeno vector + MVA-BN	Zabdeno/Mvabea (Ad26.ZEBOV + MVA-BN-Filo)	The European Medicines Agency	Anti-EBOV GP binding antibodies [264]	53.4 [233]	Not ideal for outbreak settings Pre-existing immunity to vector reduces vaccine effect Interaction and interference between vaccine and therapeutics Various inoculation may produce different immune response [233]	SAE + myalgia, Joint pain	No data available

All the adverse effect details are adapted from the fact sheet of corresponding vaccines. Ad—Adenovirus; chAd-Chimpanzee Adenovirus; EMA—European Medicine Association; GP—Glycoprotein, HPIV3—Human parainfluenza virus 3; LNP-Lipid nanoparticles; MVA—Modified vaccinia Ankara; nAb—Neutralizing Antibodies; NP—Nucleoprotein; sGP—spike Glycoprotein; S—Spike protein; N-Nucleocapsid DNA; UK-VP—UK Vaccination Program; VLPs—Virus-like particles; VSV—Vesicular stomatitis virus; rVSV—recombinant vesicular stomatitis virus; STRCT—The Scientific and Technological Research Councils of Turkey.

## Data Availability

Not applicable.

## Supplementary Materials

The following supporting information can be downloaded at https://www.mdpi.com/article/10.3390/pathogens11070800/s1, Table S1: Immunomodulatory approaches for COVID-19 and EVD, Table S2: An overview of COVID-19 and EVD vaccines under clinical trial on the basis of common vaccine platforms.
