# Supplementary material for: RNA Viruses, Pregnancy and Vaccination: Emerging Lessons from COVID-19 and Ebola Virus Disease
Source: Pathogens. 2022 Jul 15;11(7):800. doi: 10.3390/pathogens11070800 (PMC9322689; doi:10.3390/pathogens11070800)
Supplement: Supplementary file 1 [file pathogens-11-00800-s001.zip › pathogens-1766684-supplementary.pdf]

Supplementary Table S1. Immunomodulatory approaches for COVID-19 and EVD

Disease	Therapeutic Strategy	Drug	Target	Mode of administration	Status/Study details	Reference
**COVID-19**	Anti-cytokines	Anakinra	IL-1R receptor	IV, SC	NCT04603742-Phase 2, NCT04443881 & NCT04362111-Phase 3	https://link.springer.com/article/10.1007/s11010-021-04325-9 https://link.springer.com/article/10.1007/s00011-021-01445-2 https://link.springer.com/article/10.1007/s11010-021-04325-9 https://www.ncbi.nlm.nih.gov/pmc/articles/PMC7252146/
		IL-37 & IL-38	IL-18Ra receptor & Il-1b	-	In-vitro	https://www.ncbi.nlm.nih.gov/pmc/articles/PMC7252146/
		Tocilizumab	IL-6 receptor	IV	NCT04445272, NCT04317092 & NCT04412772- Phase 2	https://link.springer.com/article/10.1007/s11010-021-04325-9 https://link.springer.com/article/10.1007/s00011-021-01445-2 https://www.ncbi.nlm.nih.gov/pmc/articles/PMC7252146/ https://www.frontiersin.org/articles/10.3389/fimmu.2020.01949/full https://pubmed.ncbi.nlm.nih.gov/33946736/
		Sarilumab	IL-6 receptor	IV	NCT04315298 & NCT04327388-Phase3	https://www.ncbi.nlm.nih.gov/pmc/articles/PMC7441891/ https://www.ncbi.nlm.nih.gov/pmc/articles/PMC7441891/
	Janus Kinase (JAK) Inhibitors	Baricitinib, Baricitinib + Remdesivir	JAK1 & JAK2	Oral Tablet, Oral Tablet + IV	NCT04321993-Phase 2, NCT04358614, NCT05074420, NCT04421027 & NCT04640168-Phase 3	https://link.springer.com/article/10.1007/s11010-021-04325-9 https://www.ncbi.nlm.nih.gov/pmc/articles/PMC7252146/

						https://link.springer.com/article/10.1007/s11010-021-04325-9 https://www.frontiersin.org/articles/10.3389/fimmu.2020.01949/full https://www.ncbi.nlm.nih.gov/pmc/articles/PMC7441891/ https://pubmed.ncbi.nlm.nih.gov/33946736/
		Ruxolitinib	JAK1 & JAK2	Oral tablet	NCT04334044- Phase-2 & NCT04362137 - Phase 3	https://www.mdpi.com/1424-8247/13/8/188/htm https://www.ncbi.nlm.nih.gov/pmc/articles/PMC7441891/ https://pubmed.ncbi.nlm.nih.gov/33946736/ https://www.ncbi.nlm.nih.gov/pmc/articles/PMC7441891/
	Anti-TNF	Infliximab	TNFα	IV	NCT04425538 - Phase 2, NCT04593940 & NCT04330690-Phase 3	https://link.springer.com/article/10.1007/s11010-021-04325-9 https://www.frontiersin.org/articles/10.3389/fimmu.2020.01949/full https://pubmed.ncbi.nlm.nih.gov/33946736/
		Adalimumab	TNFα	SC	NCT04705844-withdrawn	https://pubmed.ncbi.nlm.nih.gov/33946736/
	GM-CSF	Sargramostim/ Leukine	rhuGM-CSF	Nebulization, IV	NCT04411680, NCT04569877-Phase 2 & NCT04326920- Phase 4	https://link.springer.com/article/10.1007/s40265-020-01367-z
		Gimsilumab,	GM-CSF	IV	NCT04351243-Phase 2	https://www.ncbi.nlm.nih.gov/pmc/articles/PMC7441891/
		Lenzilumab, Lenzilumab + Remdesivir	GM-CSF, IL1β	IV	NCT04583969-Phase 2 & NCT04351152- Phase 3	https://www.ncbi.nlm.nih.gov/pmc/articles/PMC7441891/

		Namilumab	GM-CSF	IV	ISRCTN40580903	https://www.ncbi.nlm.nih.gov/pmc/articles/PMC8676420/
		Mavrilimumab	GM-CSFα receptor	IV	NCT04492514, NCT04463004 & NCT04399980-Phase 2 & NCT04447469-Phase 3	https://www.ncbi.nlm.nih.gov/pmc/articles/PMC7441891/
	Convalescent plasma	Neutralizing Abs	-	IV	NCT04412486- Early phase 1, NCT04456413, NCT04384497-Phase 2 & NCT04873414-Phase 3	https://www.mdpi.com/1424-8247/13/8/188/htm<br><br>https://pubmed.ncbi.nlm.nih.gov/33946736/
	IVIG	-	T & B-cell, Fcγ receptor	IV	NCT05350774, NCT04432324 & NCT04521309-Phase 2,	https://www.ncbi.nlm.nih.gov/pmc/articles/PMC7252146/<br><br>https://pubmed.ncbi.nlm.nih.gov/33946736/
	Corticosteroids	Dexamethasone	-	IV, Oral & Intranasal	NCT04707534-Phase 4, NCT04603729, NCT04509973, NCT05293210-Phase 3 & NCT04513184-Phase 2	https://link.springer.com/article/10.1007/s11010-021-04325-9
		Methylprednisolone	-	IV	NCT04780581-Phase 4, NCT04499313, NCT04673162 & NCT04438980-Phase 3 & NCT05350774-Phase 2	https://link.springer.com/article/10.1007/s40265-020-01367-z
		**Budesonide**	ACE2 receptor	Inhalation	NCT04964414 & NCT04416399-Phase 2, NCT04361474-Phase 3 & NCT04355637-Phase 4	https://www.ncbi.nlm.nih.gov/pmc/articles/PMC8040526/<br><br>https://erj.ersjournals.com/content/59/3/2103036.abstract

		**Ciclesonide**	-	Inhalation	NCT04381364-Phase 2 & NCT04377711-Phase 3	https://journals.asm.org/doi/pdf/10.1128/JVI.01648-20
	Interferons	IFN-α2b	IFN receptor (IFNAR)	Nebulization	NCT04988217, NCT04480138 & NCT04379518-Phase 2	https://www.mdpi.com/1424-8247/13/8/188/htm<br><br>https://www.frontiersin.org/articles/10.3389/fimmu.2020.01949/full
		IFN-β1b	IFN α/β receptor (IFNAR)	Nebulization, SC	NCT04988217, NCT04343768 & NCT04647695 -Phase 2	https://link.springer.com/article/10.1007/s11010-021-04325-9<br><br>https://www.frontiersin.org/articles/10.3389/fimmu.2020.01949/full<br><br>https://www.ncbi.nlm.nih.gov/pmc/articles/PMC7445008/
		Emapalumab	IFNγ receptor	IV	NCT04324021	https://www.ncbi.nlm.nih.gov/pmc/articles/PMC7441891/
	Sphingosine-1-receptor modulator	Fingolimod	lymphocyte	Oral	NCT04280588-withdrawn & NCT04796584-recruiting	https://www.ncbi.nlm.nih.gov/pmc/articles/PMC7441891/<br><br>https://pubmed.ncbi.nlm.nih.gov/32784499/
	Monoclonal Ab	Bevacizumab	VEGF	IV	NCT04275414-Phase 2, NCT04822818-Phase 3	https://www.ncbi.nlm.nih.gov/pmc/articles/PMC7441891/
	Anti-complement therapy	Eculizumab	complement protein C5	IV	NCT04355494-no longer available & NCT04346797-Phase 2	https://www.ncbi.nlm.nih.gov/pmc/articles/PMC7441891/
	Synthetic VIP	Avipatidil	TNFα & IL-6,	IV, Inhalation	NCT04311697, NCT04536350 & NCT04844580 -Phase 2	https://link.springer.com/article/10.1007/s11356-021-17824-5
	-	Thalidomide	PGE2, TNFα, IL-1, IL-6	Not available	NCT04273529-Phase 2	https://www.ncbi.nlm.nih.gov/pmc/articles/PMC7441891/
	Macrolides	Azithromycin		Oral	NCT04329832-Phase 2, NCT04332107-Terminated, NCT04381962-Phase 3	https://link.springer.com/article/10.1007/s40265-020-01367-z

		Clarithromycin	NAADP-stimulated lysosomal calcium	Oral	NCT04398004- Phase 2, NCT04622891- not applicable	https://link.springer.com/article/10.1007/s40265-020-01367-z https://www.sciencedirect.com/science/article/pii/S0166354216306167
	-	Thymalfsin/ Thymosin α-1	T-cell	SC, Nasal drop	NCT04428008, NCT04487444-Phase 2, & NCT04320238-Phase 3	https://pubmed.ncbi.nlm.nih.gov/32442287/ https://www.frontiersin.org/articles/10.3389/fimmu.2021.673693/full
	Colchicine	-	NOD, LRR & NLRP3	Oral tablet	NCT05038449-not yet started NCT05118737-Early phase 1, NCT04355143, NCT04375202, NCT04360980-Phase 2,	https://link.springer.com/article/10.1007/s40265-020-01367-z
	Statins	Simvastatin	MyD88	Oral	NCT04348695- Phase 2	https://link.springer.com/article/10.1007/s11010-021-04325-9 https://www.ncbi.nlm.nih.gov/pmc/articles/PMC7252146/
	Selective estrogen receptor modulators (SERMS)	Clomiphene	NPC1	-	In-vitro	https://www.ncbi.nlm.nih.gov/pmc/articles/PMC7578196/
	Pan-Caspase inhibitor	Q-VD	caspase	-	Invitro study	https://www.nature.com/articles/s41418-022-00936-x
**EVD**	Monoclonal Abs	ZMapp	GP	IV	NCT02363322-Phase 1, NCT03719586-Phase 2	https://pubmed.ncbi.nlm.nih.gov/28890666/ https://www.ncbi.nlm.nih.gov/pmc/articles/PMC6498552/
		mAb114	VRC608	IV	NCT03478891- Phase 1, NCT03719586- phase 3	https://www.sciencedirect.com/science/article/pii/S0140673619300364 https://www.nejm.org/doi/full/10.1056/NEJMe1915350 https://www.nejm.org/doi/full/10.1056/NEJMe1915350

		REGN-EB3	GP	IV	NCT03719586Phase 2/3	https://www.nejm.org/doi/full/10.1056/NEJMe1915350
		α-IL-6R mAbs	Il-6	IV	In-vivo	https://www.frontiersin.org/articles/10.3389/fphar.2020.574703/full
		Gamezumab	-	-	NCT04717830-Phase 1	https://clinicaltrials.gov/ct2/show/NCT04717830?term=Gamezumab&cond=ebola&draw=2&rank=1
	RNA synthesis	TKM-Ebola		IV	NCT01518881-Terminated	https://pubmed.ncbi.nlm.nih.gov/25630412/<br><br>https://www.ncbi.nlm.nih.gov/pmc/articles/PMC6498552/
	Macrolides	Azithromycin	-	IV	NCT02380625- Phase 1	https://link.springer.com/article/10.1007/s40506-017-0130-z
	Topoisomerase II Inhibitors	Doxorubicin	IFN	-	Invitro study-cell line	https://journals.asm.org/doi/pdf/10.1128/mBio.00368-17
	Interferons	IFN-γ	IFN-γ receptor (IFNGR)	-	Invitro study-mouse cell line	https://journals.plos.org/plospathogens/article?id=10.1371/journal.ppat.1005263<br><br>https://journals.plos.org/plospathogens/article?id=10.1371/journal.ppat.1005263
		IFN-α & β	IFN α/β receptor (IFNAR)	-	invitro	https://academic-oup-com.proxy.lib.duke.edu/jid/article/179/Supplement_1/S188/881048<br><br>https://www.ncbi.nlm.nih.gov/pmc/articles/PMC5743218/
		Neplanocin A, 3-deazaneplanocin A	IFNs		In-vivo	https://www.ncbi.nlm.nih.gov/pmc/articles/PMC7110990/
	Covalent plasma	Neutralizing Abs	Whole virus/GP	IV	Treatment in Guinea	https://pubmed.ncbi.nlm.nih.gov/28890666/<br><br>https://www.ncbi.nlm.nih.gov/pmc/articles/PMC6498552/<br><br>https://www.nejm.org/doi/10.1056/NEJMoa1511812<br><br>https://link.springer.com/article/10.1007/s40506-017-0130-z

	Agonist	Eritoran	TLR	-	In-vivo	https://journals.asm.org/doi/10.1128/mBio.00226-17
	Nucleic acid analog	Favipiravir (T705)	RNA polymerase	Oral	NCT02662855 Phase 2	https://www.ncbi.nlm.nih.gov/pmc/articles/PMC6498552/<br><br>https://pubmed.ncbi.nlm.nih.gov/33068263/<br>https://www.sciencedirect.com/science/article/pii/S0166354214000576
		BCX4430 (galidesivir)	Viral RNA polymerase	IM	NCT02319772<br>Phase 1	https://link.springer.com/article/10.1007/s40506-017-0130-z
		GSK983	dihydroorotate dehydrogenase	-	In-vitro	https://www.ncbi.nlm.nih.gov/pmc/articles/PMC6436837/
	Comb. Antisense phosphorodiamidate morpholino oligomers	AVI-6002	VP24 and vP35	IV	NCT01353027<br>Phase 1	https://pubmed.ncbi.nlm.nih.gov/25630412/
	Selective estrogen receptor modulators (SERMS)	Clomiphene	VLPs and trVLPs	-	In-vitro	https://www.ncbi.nlm.nih.gov/pmc/articles/PMC7110990/<br><br>https://mdpi-res.com/viruses/viruses-08-00206/article_deploy/viruses-08-00206.pdf?version=1470137492
	Indoline-based alkaloid-like derivative	CMLDBU3402		-	-	https://pubmed.ncbi.nlm.nih.gov/25532798/
	Viral entry fusion modulators	E-64d, E-64	$GP_{1,2}$	-	In-vitro	https://pubmed.ncbi.nlm.nih.gov/25532798/
		Nilotinib & imatinib	c- Abl1 kinase	-	In-vitro	https://pubmed.ncbi.nlm.nih.gov/25532798/
		Okadaic acid	PP1 & PP2A,	-	In-vitro	https://pubmed.ncbi.nlm.nih.gov/25532798/
	Endoplasmic reticulum (ER) glucosidase inhibitors	IHVR11029, IHVR17028 & IHVR19029	ER a-glucosidases I & II	-	In-vitro	https://www.ncbi.nlm.nih.gov/pmc/articles/PMC7110990/<br><br>https://pubmed.ncbi.nlm.nih.gov/25532798/
	Lectins	vacuolin-1	V-ATPase & RAB5A GTPase r	-	-	https://pubmed.ncbi.nlm.nih.gov/25532798/
		Bafilomycin A1, cyanovirin N & concanamycin A,	V-ATPase	-	In-vitro	https://pubmed.ncbi.nlm.nih.gov/25532798/

						https://www.ncbi.nlm.nih.gov/pmc/articles/PMC7110990/

ECMO-extracorporeal membrane oxygenation, VEGF - angiogenic vascular endothelial growth factor, VIP - Vasoactive Intestinal polypeptide- PGE2 - Prostaglandin E2, IFN- Interferons, Monoclonal Abs- Monoclonal antibodies, IVIG -Intravenous Immunoglobulin G, rhuGM-CSF- yeast-derived recombinant humanized GM-CSF, ISG- Interferon stimulated gene, IV-Intravenous, IM-Intramuscular, IP-Intraperitoneal, SC-subcutaneous, TLR4- toll like receptor, PP1- protein phosphatases 1, PP2A- protein phosphatases 2A

Supplementary Table S2. An overview of COVID-19 and EVD vaccines under clinical trial based on common vaccine platforms.

Vaccine platform	Disease	Commercial name	Name of company	Immunogen	Phase of vaccine development/Clinical trial Identifier number
DNA	COVID-19	ZyCoV-D	Zydus Cadila, Cadila Healthcare Ltd, India	S +IgE	Phase 3, CTRI/2021/01/030416
		INO-4800	Inovio Pharmaceuticals, International Vaccine Institute	S	Phase 3, NCT04336410
		AG0301-COVID19	AnGes Inc., Osaka University, Takara Bio	S	Phase 3, NCT04463472
		Covigenix VAX-001	Entos Pharmaceuticals Inc.	S	Phase 2, NCT04591184
		COVID-eVax	Takis	S	Phase 2, NCT04788459
		VB10.2210	Nykode Therapeutics	S & non-S T-cell	Phase 2, NCT05069623
		VB10.2129	Nykode Therapeutics	RBD	Phase 2, NCT05069623
		GLS-5310	GeneOne Life Science Inc	S	Phase 1, NCT04673149
		bacTRL-Spike	Symvio	S	Phase 1, NCT04334980
		COVIGEN	University of Sydney	S	Phase 1, NCT04742842
		COVIDITY	Scancell	S, N	Phase 1, NCT05047445
		CorVax12	Providence Health & Service	S	Phase1, NCT04627675
	EVD	INO-4212/SynCon (INO-4201 + INO-4202)	Inovio Pharmaceuticals	GP, NP	Phase 1, NCT02464670
ChAd	COVID-19	AZD 1222 (ChAdOx1 nCoV-19)	AstraZeneca/Oxford University	S	Phase 3, NCT05126992
		Covishield (ChAdOx1_nCoV19)	Serum Institute of India	S	Phase 3, NCT04794946
		ChAdV68-S	NIAID	S	Phase 1, NCT04776317
	EVD	ChAd3-EBOZ with or without Mvabea (cAd3-ZEBOV; ChAd3-EBO-Z)	GlaxoSmith Kline Okairos & NIAID, Sabin Vaccine Institute	GP	Phase 1/2, NCT03583606
		chAd3-Marburg	Sabin Vaccine Institute	GP	Phase 1, NCT03475056
Ad26	COVID-19	Janssen (Ad26.COV2. S)	Johnson & Johnson (Janssen Biotech Inc.)	S	Phase 3, NCT05220397
		SC-Ad6-1	Tetherex Pharmaceuticals Co.	S	Phase 1, NCT04839042
		Sputnik/ Gam-COVID-Vac (rAd26 + Ad5)	Gamaleya Research Institute of Epidemiology and Microbiology	S	NCT04642339
		Spuitnik Light	Gamaleya Research Institute of Epidemiology and Microbiology	S	Phase 3, NCT04741061

	EVD	Zabdeno/Mvabea (Ad26.ZEBOV + heterologous MVA-BN-Filo)	Johnson & Johnson (Janssen Biotech Inc.)	GP, NP	Phase 3, NCT05064956
Ad5	COVID-19	Convidecia (Ad5-nCoV)	CanSino Biologics	S	Phase 3, NCT05313646
		hAd5-Covid19	Immunity Bio Inc	S & N	Phase 2, NCT04845191
		VXA-CoV2-1.1-S	Vaxart	S & N	Phase2, NCT05067933
		AdCLD-CoV19	Cellid Co	S	Phase 2, NCT05047692
		Ad5-triCoV/Mac	McMaster University	S	Phase 1, NCT05094609
	EVD	Ad5-EBOV	Beijing Institute of Biotechnology & CanSino Bio	GP	Phase 2, NCT02533791
Protein subunit	COVID-19	NVX-CoV2373 (Nuvaxoid)	Novavax (Gaithersburg, USA)	S-RBD	Phase 3, NCT05249816
		Covovax	Serum Institute of India	S-RBD	Phase 3, ictrp-CTRI202204042017
		TAK-019	Takeda, Tokyo, Japan (based on Novavax formulation)	S	Phase 3, NCT05299359
		Abdala	Center for Genetic Engineering and Biotechnology	S	Phase 3, ictrp-RPCEC00000359
		EpiVacCorona	Vector State Research Center for Virology and Biotechnology, Russian Federation	S	Phase 3, NCT04527575
		Corbevax	Biological E Limited, Hyderabad, India	S	Phase 3, ictrp-CTRI202108036074
		ZifiVax (ZF2001/ZF-UZ-VAC 2001)	Anhui Zhifei Longcom, Beijing, People's Republic of China	S-RBD	Phase 3, NCT04646590
		Noora Vaccine	Bagheit-allah University of Medical Sciences	S	Phase 3, ictrp-IRCT20210620051639N3
		MVC-COV1901	Medigen, Taipei, Taiwan	S	NCT04695652
		Aurora-CoV (EpiVacCorona-N)	Vector State Research Center for Virology and Biotechnology, Russian Federation	S	Phase 2, NCT04527575
		Soberana Plus (FNILAY -FR-1A)	Institute Finaly de Vacunas	S	Phase 3, RPCEC00000374
		MVC-COV1901	Medigen	S-2P	Phase 3, NCT04695652
		Razi Cov Pars	Razi Vaccine and Serum Research Institute	S	Phase 3, ictrp-IRCT20201214049709N5

		Sponge (COVAX-19)	Vaxine/CinnaGen Co	S	Phase 3, NCT04453852
		EVD Filovirus glycoprotein	University of Hawaii, BIOQUAL Inc., Galveston National Lab	GP	Pre-clinic/NHP, NCT05079750
VSV	COVID-19	Brilife/IIBR-100 (VSV-ΔG SARS-CoV-2)	Israel Institute for Biological Research	S	Phase 3, NCT04990466
	EVD	Ervebo (rVSVΔG-ZEBOV/, rVSVΔG-ZEBOV-GP)	Merck NewLink Genetics PHAC (National Microbiology Laboratory in Winnipeg)	GP	Phase 3, NCT05202288
		GamEvac-Combi and GamEvacLyo (Heterologous rVSV and Ad5)	Gamaleya Research Institute of Epidemiology and Microbiology (Russia)	GP	Phase 4, NCT02911415
VLPs	COVID-19	LYB001	Yantai Patronus Biotech Co Ltd	-	Phase 3, NCT05137444
		SARS-CoV-2 VLP Vaccine	The STRCT	VP	Phase 2, NCT04962893
		VBI-2902a	VBI Vaccine Inc.	S	Phase 2, NCT04773665
Nanoparticles	COVID-19	COVID-19-EDV	EnGeneIC	S	Phase 1, ACTRN12621001159842
		SAM-LNP-S	NIAID	S	Phase 1, NCT04776317
